# Health protective behavior scale: Development and psychometric evaluation

**DOI:** 10.1371/journal.pone.0190390

**Published:** 2018-01-08

**Authors:** Weiwei Ping, Wenjun Cao, Hongzhuan Tan, Chongzheng Guo, Zhiyong Dou, Jianzhou Yang

**Affiliations:** 1 Department of Preventive Medicine, Changzhi Medical College, Changzhi, Shanxi, P. R. China; 2 Department of Epidemiology and Health Statistics, School of Public Health, Central South University, Changsha, Hunan, P. R. China; 3 Health Check Centre, Heping Hospital Affiliated to Changzhi Medical College, Changzhi, Shanxi, P. R. China; Public Library of Science, UNITED KINGDOM

## Abstract

**Objective:**

A healthy lifestyle includes health protective and health promoting behaviors. Health promoting lifestyle profiles have been developed, but measures of health protective behavior are still lacking. This study sought to develop a health protecting behavior scale.

**Methods:**

An initial item pool for the Health Protective Behavior Scale (HPBS) was generated based on read and referred literature and a single-item open-ended survey. An expert group screened this initial item pool using an item-level content validity index. Pilot testing was conducted. The degree of variation, the response rate, the item-total correlation coefficient, and the factor loading in factor analysis and item analysis were used to screen items using data of pilot testing. 454 subjects were recruited evaluate the psychometric properties of the HPBS. Analyses included internal consistency, test-retest reliability, factor analysis, parallel analysis, correlation analysis and criterion validity analysis.

**Results:**

The final iteration of the HPBS was developed with 32 items and five dimensions: interpersonal support, general behavior, self-knowledge, nutrition behavior and health care. Cronbach’s alpha coefficient, and test-retest reliability were 0.89 and 0.89 respectively. Correlation coefficients of the five dimensions ranged from 0.28 to 0.55. The Spearman correlation coefficient between the total scores on the WHOQOL-BREF and on the HPBS was 0.34.

**Conclusions:**

HPBS has sufficient validity and reliability to measure health protective behaviors in adults.

## Introduction

In recent decades, public health specialists around the world have been emphasizing the importance of healthy lifestyle [1–2]. At least 60% of the burden of diseases around the world is due to unhealthy lifestyles, according to World Health Organization (WHO) reports [3]. The harm of unhealthy lifestyle activities such as smoking, excess alcohol and fat consumption, lack of exercise, and chronic exposure to environment pollutants has been paid close attention, and has been documented in many studies [4–8]. In contrast, changing an unhealthy lifestyle and establishing a healthy one can improve individuals’ health conditions and prevent or decrease many diseases; this also has been established by many studies [9–10]. In sparking healthy lifestyles, the first challenge is how to define and measure healthy lifestyle-related behaviors.

Pender suggested that health-protecting and health-promoting behavior might be viewed as complementary components of healthy lifestyle, and suggested that health–promoting behavior, an expression of the human actualizing tendency, was directed toward sustaining or increasing the individual’s level of well-being, self-actualization, and personal fulfillment [11]. Walker developed the Health-Promoting Life-style Profile (HPLP), which was portrayed as a multi-dimensional pattern including self-actualization, health responsibility, exercise, nutrition, interpersonal support, and stress management [12]. In 1995, the HPLP was revised as HPLP-Ⅱ. Thirty-one of the original items were retained, while 21 items were added or underwent major revision [13]. The HPLP has been used widely to evaluate lifestyle changes, including cross-cultural influences on health behavior [14–16].

Harris defined health protective behavior (HPB) as any behavior performed by a person, regardless of his or her perceived or actual health status, to protect, promote, or maintain his or her health, whether or not such behavior is objectively effective toward health[17]. Pender suggested that HPB should be viewed as an expression of the human stabilizing tendency and directed toward decreasing the individual’s probability of encountering illness [11]. Walker had tried to measure HPB together with health promoting behavior in a single profile, but the items concerned with HPB were eliminated on the basis of item analysis, which showed that HPB is different from what is measured on the HPLP.

Some investigators have measured health related behaviors that focus on quality of life, e.g., Crispin’s 1993 Short Form 36 (SF-36) [18]. Bull developed the Global Physical Activity Questionnaire (GPAQ) [19], as a measure of physical activity. Tran DV created the International Physical Activity Questionnaire (IPAQ) [20], a standardized instrument for physical activity monitoring among older adults. Lotfi constructed an instrument to predict the protective sexual behaviors in women at risk of human immunodeficiency virus [21]. All of these instruments emphasized physical health, psychological health or a particular aspect of health, but had no focus on health protective behavior. Therefore, it is essential to develop a valid and reliable instrument to measure health protective behavior.

Health protective behavior encompasses multiple dimensions, which may include the four aspects of environment, behavior and lifestyle, genetic factors, and health care [22]; or may be expressed by eight key factors, namely, of safety, social security, education, food security, income, ecological environment, sustainable resources, and social justice [23]. Our development of a health protective behavior scale involved selection of items across these dimensions to represent the health protective behaviors of adults 18–59 years old.

This article described the development and initial psychometric evaluation of the Health Protective Behavior Scale (HPBS). The study including two steps: in step 1, the HPBS was developed; in step 2, its psychometric properties, including validity and reliability, were evaluated.

## Materials and methods

### Step 1: HPBS scale development

#### 1. Read and referred literatures

To help select the candidate items reflecting health protective behavior, we read literatures coming from Medline, Embase, China National Knowledge Infrastructure, Wangfang Data, and Weipu data, and obtained content related to health protective behavior. Short form (SF-36)[18], WHO Quality of Life Scale (WHOQOL-100)[24], Social Support Rate Scale(SSRS)[25], Health-Promoting Life-style Profile-Ⅱ (HPLP-Ⅱ)[13], Global Physical Activity Questionnaire (GPAQ)[19], Nottingham Health Profile(NHP) [26]were read in detail, and some items were referenced for health protective behavior scale.

#### 2. Open-ended survey

An open-ended survey was conducted in Mar, 2014; the survey question was “what are you doing to protect your health?”

The subjects were recruited from a city located in Shanxi Province, China, with a population of more than 350,000. The study met relevant ethical guidelines. All subjects in this research agreed to participate in this study, and signed a written informed consent. The protocol was approved by Changzhi Medical College Ethics Review Committee.

A two-stage stratified sampling method was applied. First, three communities (Huaihai, Dongjie, Xinganquan) were selected randomly from the eight communities of the city. Second, five buildings were selected randomly from each of the three communities. All individuals who resided in the chosen buildings were invited if they met the following criteria: (1) aged between 18 to 59 years; (2) disease-free according to self-report; and (3) able to read normally. By combining the results of the literature review and the open-ended survey, we established an initial pool of 96 items.

#### 3. Expert group

The initial item pool was screened for content validity by a group of 12 experts including two psychologists, two health educators, two sociologists, two doctors, one nursing faculty, one dietitian and two epidemiologists. The content validity index (CVI) reflects the correlation of variables with a conceptual definition [27]. The expert group independently ranked each item in the pool as follows: 1 = not relevant; 2 = somewhat relevant; 3 = quite relevant; and 4 = highly relevant [28]. An item-level content validity index (I-CVI) was computed, wherein the values of I-CVI for an item is the proportion of experts who ranked the item as 3 or 4. An I-CVI above 0.78 was defined as having good content validity [29]; items that met this criterion constituted the pilot version of the HPBS. Items with I-CVI values below 0.78 were deleted.

#### 4. Pilot testing and item screening

Pilot testing was conducted with a random sample selected from the same communities as in the opening survey (but not the same subjects) in May, 2014. The questionnaire included descriptive information and the pilot version of the HPBS. Descriptive information comprised sociodemographic characteristics of respondents (age, gender, education level, marital status, income per month, occupation). To maximize the response rate and avoid researchers’ influence on the respondents, all questionnaires were delivered and collected face-to-face by community staff that had been trained as interviewers. Respondents completed questionnaires individually, and interviewers could explain any unclear questions without influencing the subjects’ responses. All questionnaires no less than 90% of items answered were entered EpiData 3.0 for further analysis.

An item was deleted when it met any of the following three criteria: (1) the degree of variation was above 0.3; (2) the response ratio was less than 95%; (3) the item-total correlation was less than 0.3; (4) the factor loading was less than 0.4 in factor analysis; or (5) the critical ration (*t*-value) was less than 3.00 in item analysis.

### Step 2: Evaluate psychometric properties of HPBS

#### 5. Participants and contents

The psychometric properties of the final version of the HPBS was evaluated in Sept, 2014,which invoved another random sample selected from the same communities (but not the same subjects) as in the open-ended survey. To evaluate the reliability of the results, we randomly selected about 10% of the total respondents to complete a retest 14 days after the first test. The questionnaire included three parts: (1) general demographic data; (2) the HPBS; and (3) the WHOQOL-BREF in Chinese. The profile of the WHOQOL-BREF demonstrated good reliability and validity in Fang’s evaluation performed in 1999 in China [24]. The protocols for delivering and collecting questionnaires, for answering questions from subjects, and for retaining or deleting items and entering data were all identical to those described for the pilot phase.

#### 6. Data analysis

All collected data were entered into Epidata3.0 software. SPSS version 19.0 was used to analyze the data. All hypothesis tests were two-tailed with a type I error rate fixed at 0.05.

The internal consistency of the HPBS items was assessed by Cronbach’s alpha. A coefficient above 0.70 was considered satisfactory [30]. Test-retest reliability was examined using the Intraclass Correlation Coefficient (ICC). Construct validity was assessed by correlation analysis, principal component factor analysis using varimax rotation with Kaiser Normalization and parallel analysis was used to determine the number of factors to retain[31,32]. Criterion-related validity [33] was assessed by Spearman correlation coefficient (*r*) between the total scores on the WHOQOL-BREF and on the HPBS. Correlations were categorized as low (<0.40), moderate (0.40–0.59), substantial (0.60–0.79), and high (>0.80) [34,35].

Missing values were managed as follows: if two or more items in one dimension were missing, the subject’s score on this dimension was excluded from the statistical analysis; if just one item was missing, the mean value of that dimension for that subject was used to impute the missing values. The raw score for each of the HPBS dimensions was derived by summing the item scores; and this score was then converted to a standardized score, which is as follows [18]:

$$\mathrm{standardized}\;\mathrm{scale}=\frac{\mathrm{actual}\;\mathrm{raw}\;\mathrm{score}\;\text{-}\;\mathrm{lowest}\;\mathrm{possible}\;\mathrm{raw}\;\mathrm{score}}{\mathrm{possible}\;\mathrm{raw}\;\mathrm{score}\;\mathrm{range}}\times 100$$

#### 7. Ethical statement

The work met relevant ethical guidelines. Written informed consent was obtained from all subjects of this research, in accordance with the guidelines of Changzhi Medical College Ethics Review Committee. The protocol was approved by Changzhi Medical College Ethics Review Committee.

## Results

### HPBS development

An initial pool of 96 items was established by literature review and open-ended survey conducted with 201 subjects. Table 1 shows the general framework of the initial item pool of the HPBS.

**Table 1:** General framework of the initial item pool of the HPBS.

Possible aspect	Related behaviors
Environment	occupation health, living condition, public health, food safety, drinking water, air pollution, hygiene habits
Lifestyle	diet(fruit, vegetable, sugar, salt, fat), physical activity, drink, smoking, driving
Genetic factors	screening disease, intermarriage, vaccination
Health care	accessibility of health care, pharmacy behavior, behavior of following doctor's instruction, sexual behavior, reproductive health
Mental health	relaxation, coping with stress, ask for help
Social factor	traffic safety, waste disposal, medical security

After the expert assessment, 36 items remained in the pilot version of the HPBS (CVI 0.78–1.00) and 60 items were deleted (CVI<0.78). The 36 candidate items were divided into two categories: 29 items with hierarchical options (1 = never, 2 = rarely, 3 = sometimes, 4 = usually, 5 = always), and 7 items with dichotomous options (1 = yes, 2 = no).

211 subjects completed the pilot version of the HPBS in pilot testing, of these, 196 (92.89%) completed it sufficiently for their data to be analyzed. All the subjects were Chinese. Their ages ranged from 18 to 59 years, with a mean age of 40.64±9.52 years; 52.13% were male and 47.92% were female. According to the criteria mentioned previously, four items were deleted, including “live in safe surroundings,” “know how to make an emergency call,” “use range hood when cooking,” and “do my best to resolve difficulties”. The remaining 32 items constituted the final HPBS version.

### HPBS evaluation

502 subjects were selected to complete the final HPBS version for the purpose of evaluating its psychometric properties; of these, 454 (90.44%) completed it sufficiently to have their data included for analysis. All of them were Chinese, their age ranged from 18 to 59 years, 55.33% were male and 44.67% were female. The HPBS has a possible score range of 32 to 145; however, the actual range of our subjects was from 32 to 125. The total mean score was 66.63, and the median was 66.01. Detailed information is shown in Table 2.

**Table 2:** Distribution of the HPBS scores on each dimension.

Factor label	Range of theoretical scores(min, and max)	Range of actual scores (min, and max)	*x*±s
Interpersonal support	(8,40)	(8,40)	17.36±5.47
General behavior	(7,35)	(7,32)	17.08±4.42
Self- knowledge	(6,15)	(6,15)	8.71±2.11
Nutrition behavior	(5,25)	(5,24)	12.25±3.70
Health care	(6,30)	(6,27)	11.38±4.00
Total	(32,145)	(32,125)	66.55±15.05

#### 1. Factor analysis and paralled analysis

**Fig 1:**
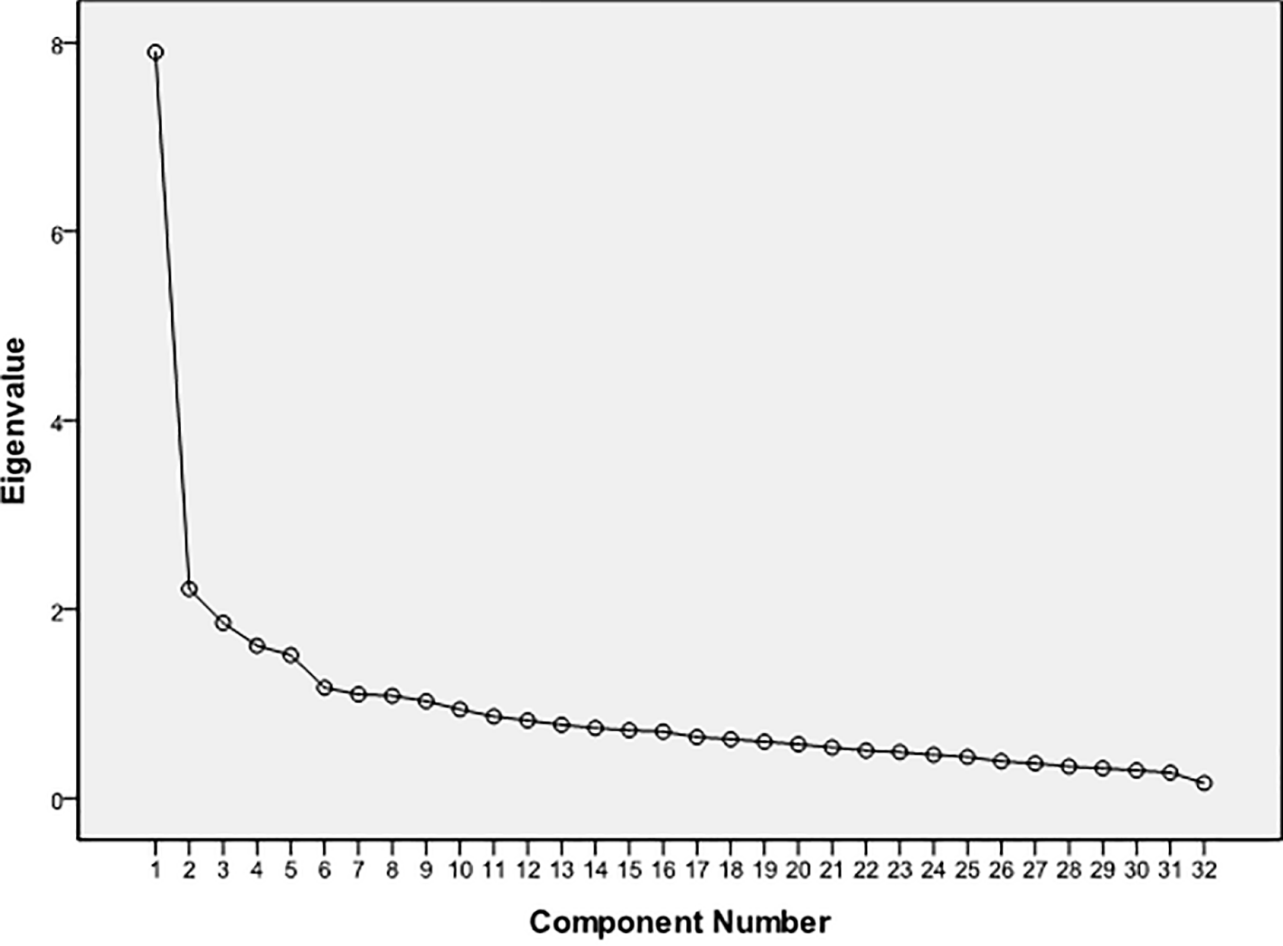
Plot of eigenvalues using factor analysis.

**Fig 2:**
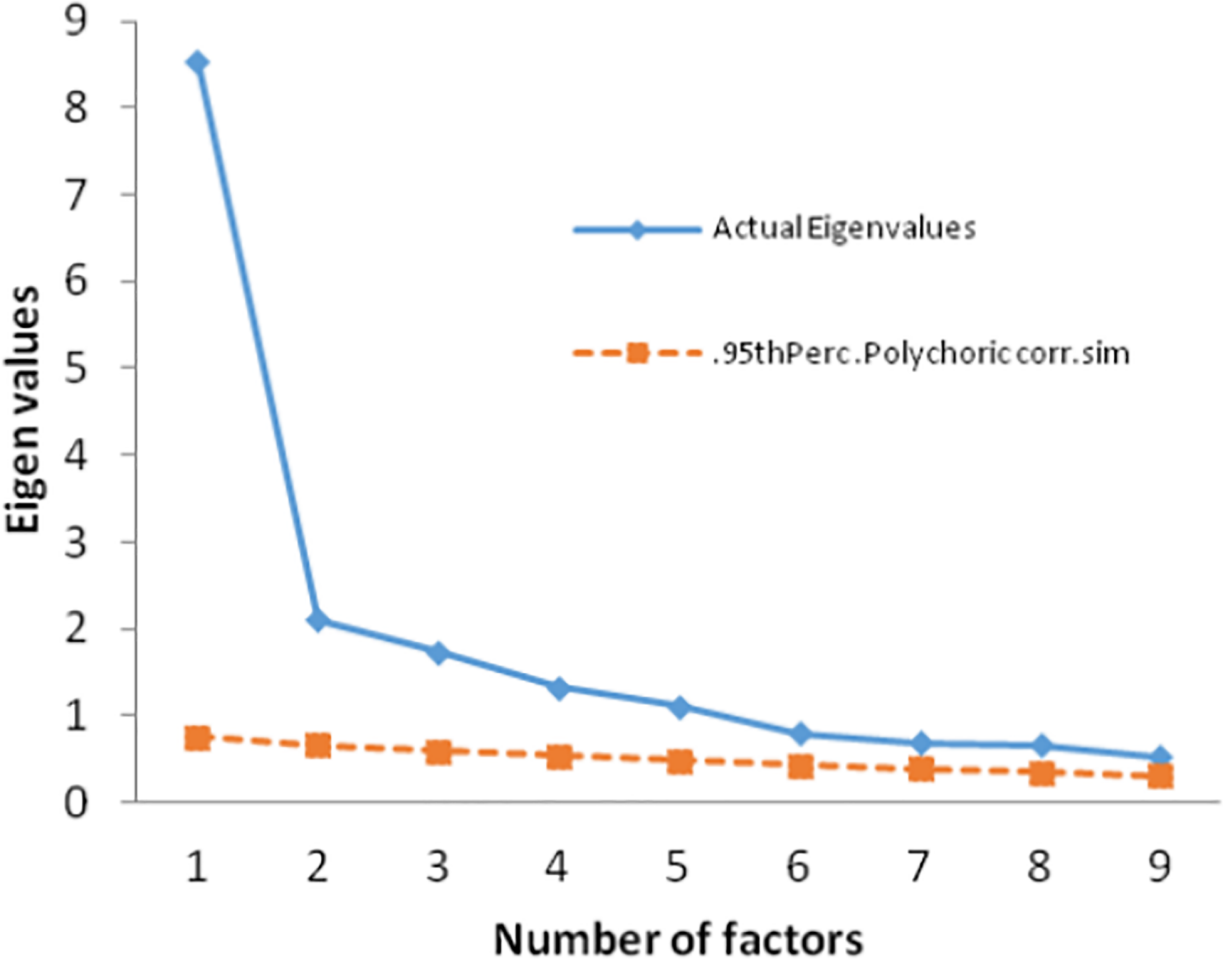
Plot of actual and randomly-generated eigenvalues of paralled analysis.

Before the exploratory analysis, Kaiser–Meyer–Olkin (KMO) and Bartlett’s test of sphericity were used to measure the appropriateness of the sample. The KMO value was 0.88, and there was statistically significant of Bartlett’s sphericity (χ^2^ = 5481.70, *df* = 630, *p* < 0.001), indicating that the samples met the criteria for factor analysis. Principal component factor analysis was performed using varimax rotation with Kaiser Normalization. Factor analysis yielded a 9-factor solution with an explained loading of 60.85% and eigenvalues >1. According to the screen plot (Fig 1), the slope of the curve became smooth at the fifth point, and factors 6 through 9 only contributed 13.67% of the accumulated variance. All items loaded on expected factors, and the loading was 0.40 or above for most items, with only five items below 0.40 (Table 3). A parallel analysis using randomly simulated polychoric correlation was used to identify the number of factors [31]. In the present study, to arrive at a set of random data eigenvalues, the original data set was randomly permuted 50 times. Then the 95th percentiles of the random data eigenvalues were compared to the real data eigenvalues [32]. The resulting plot of actual and randomly-generated eigenvalues appears in Fig 2. It is apparent that the first five eigenvalues extracted from the actual sample data outperformed those based upon simulated data. Consequently, this parallel analysis performed on the 32-item data supports a five-factor structure. Factor analysis and parallel analysis all proved the same outcome. So, the 32-item instrument yielded a five-factor solution with an explained loading of 47.18%.

**Table 3:** Factor loading and factor structure of the HPBS.

Code	Items	F1: interpersonal support	F2: general behavior	F3: Self-knowledge	F4: nutrition behavior	F5: health care
1	enjoy the pleasure at free time	0.72				
2	self-relaxation	0.72				
3	get help from others	0.64				
4	take other’s advice pleasureably	0.71				
5	keep calm in key moment	0.71				
6	do something to change anxiety	0.68				
7	try best to solve problems	0.69				
8	easily adapt to a new environment	0.66				
9	be far from smoking		0.78			
10	persuade other to quit smoking		0.75			
11	protect skin under sunshine		0.63			
12	wear a mask in the hazy or wind weather		0.51			
13	eat fruit every day about 250g		0.45			
14	get enough sleeping		0.39			
15	do physical activity every day 30 min or more		0.38			
16	do physical examination regularly			0.68		
17	know the value of blood sugar			0.62		
18	Income enough for general consumption			0.58		
19	learn method coping with disaster and emergency			0.57		
20	know the value of blood pressure			0.53		
21	use water purifying plant			0.38		
22	control salt				0.71	
23	control sugar				0.68	
24	replacing animal fat with vegetable oil				0.58	
25	eat vegetable every day about 250g-500g				0.55	
26	keep weight				0.39	
27	know harm about intermarriage					0.73
28	discard drug out of date					0.57
29	take doctors guide for medicine					0.56
30	use seat belt					0.48
31	use protective measures in workplace					0.45
32	worry for food safety					0.34
Eigenvalue		4.85	2.98	2.54	2.39	2.31
Variance explained (%)		15.17	9.33	7.93	7.47	7.28
Cumulative percentage %		15.17	24.50	32.43	39.90	47.18

Factor 1 was the strongest factor, explaining 15.17% of the loading of the HPBS. This factor reflected interpersonal support, and it included eight items: easily adapt to a new environment, enjoy the pleasure of free time, self-relaxation, get help from others, take other’s advice pleasantly, keep calm in some key moment, do something to change anxiety, and try one’s best to solve problems. Factor 2 had seven items, mainly concerned with one’s general day-to-day behavior; it was the “general behavior” factor accordingly, and accounted for 9.32% of the loading. Factor 3 had six items, was named “self-knowledge,” and was concerned mainly with one’s own health condition and learning health protecting knowledge. This factor accounted for 7.93% of the loading. Factor 4 had five items, was named “nutrition behavior,” and addressed controlling intake of certain foods (e.g., salt, sugar) and keeping certain good habits (e.g., healthy weight, replacing animal fat with vegetable oil, eating vegetables). This factor accounted for 7.47% of the loading. Factor 5 had six items, accounted for 7.23% of the loading, and was labeled “health care.” These items mainly concerned asking for help from doctors and using occupation-related protective measures (Table 3).

#### 2. Correlation analysis

The correlations among the five factors are presented in Table 4. The between-factor correlations varied from 0.26 to 0.55, and the correlations between each factor and the total score on the HPBS ranged from 0.45 to 0.81. These correlations suggest that each factor represented a distinct dimension and that there was low redundancy among dimensions.

**Table 4:** Correlations among factors in the HPBS (N = 454).

Factor label	1	2	3	4	5
Total	0.81	0.80	0.45	0.73	0.74
1 interpersonal support	1.00	0.48	0.28	0.45	0.55
2 general behavior		1.00	0.31	0.50	0.44
3 self- knowledge			1.00	0.31	0.26
4 nutrition behavior				1.00	0.40
5 health care					1.00

#### 3. Criterion validity

Criterion validity was assessed by correlating the HPBS to the WHOQOL-BREF. The Spearman coefficient between the total scores on the WHOQOL-BREF and on the HPBS was 0.34 (*p*<0.001); i.e., subjects with higher scores on the HPBS also had a significant tendency to score higher on the WHOQOL-BREF.

#### 4. Cronbach’s alpha coefficient

Cronbach’s alpha was calculated as a measure of internal consistency of the HPBS. The alpha coefficient of the five dimensions ranged from 0.64 to 0.88 (Table 5), which indicated a high internal consistency.

**Table 5:** Cronbach’s alpha coefficients of the HPBS.

Factor label	Cronbach’s alpha coefficient
Interpersonal support	0.88
General behavior	0.71
Self- knowledge	0.71
Nutrition behavior	0.71
Health care	0.64

#### 5. Test-retest reliability

To evaluate stability, the HPBS was administered twice to a sub-sample of 57 subjects at an interval of two weeks. The ICC was 0.89 for the total scale, and ranged from 0.44 to 0.85 for all items, with the exception of two items: “eat vegetables every day” (0.39) and “learn methods for coping with disaster and emergency” (0.28).

## Discussion

Healthy lifestyles include both health protective and health promoting behaviors. A health promoting lifestyle profile has been developed by Walker, and the present study developed a health protecting behavior scale. The adult period is an important stage for protecting life [36]; therefore, we chose people between 18 and 59 years as subjects. Health protective behavior on other age groups should be studied in the future.

Although the pilot HPBS identified six separate but related dimensions, only five dimensions were supported by the factor analysis and reliability estimates. Each of the five dimensions is conceptually equivalent to one of the original six dimensions or a combination of two. Only genetic factors does not appear as a factor in the HBPS. The possible reason was that although genetic factors are important for health, they cannot be changed by any health protective behavior. Other published scales have had similar trajectories; i.e., dimensions designed originally did not equate with the final dimensions [37].

Our study used the WHOQOL-BREF to assess the criterion-related validity of the HPBS. This instrument has also been used in other health measure-related studies [38–40]. The correlation of the HPBS with the WHOQOL-BREF was significant and similar to that found in other studies [41]. Because good health protective behaviors contribute to a better quality of life, the WHOQOL-BREF appears to be a reasonable measure for evaluating the criterion validity of the HPBS.

The study showed an acceptable Cronbach’s alpha value (≥0.70) for all dimensions except health care, indicating that the internal consistency of HPBS is satisfactory. The health care dimension’s relatively low alpha coefficient was mirrored in the ICC. This may be because of some problems in the conceptualization of health care or because of the large age range of our subjects, as different age groups have different considerations regarding health care behavior.

The HBPS demonstrated good test-retest reliability, with a total ICC of 0.89, and satisfying internal consistency. Only a few items had lower test-retest reliability values, such as “eat vegetables every day,” and “learn methods for coping with disaster and emergency.” These results are consistent with other studies [18, 19].

This study had some limitations. First, we used a cross-sectional design to evaluate the HPBS, which does not allow examination of its discriminant validity. Second, our sample included only subjects from Changzhi, which might limits the generalizability of the findings. So, it is necessary to apply the scale to other populations for further testing it`s items in the future. Third, as an important part, confirmatory factor analysis isn't be conducted in our study. So, it is necessary to apply the scale to another sample to evidence its construct validity in the future.

## Conclusions

The HPBS has sufficient validity and reliability. It is helpful for researchers intend to investigate the health protecting component of lifestyles in adults, and to measure changes in health protective behavior.

## Supporting information

S1 File Questionnaires. (DOC)

S2 File Data. (SAV)
