# Supplementary material for: Health protective behavior scale: Development and psychometric evaluation
Source: PLoS One. 2018 Jan 8;13(1):e0190390. doi: 10.1371/journal.pone.0190390 (PMC5757924; doi:10.1371/journal.pone.0190390)
Supplement: S1 File — (DOC) [file pone.0190390.s001.doc]

调查表

A survey in general population, Changzhi, China

| G0001 | 调查点代码:  Surveyed site number: | 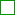 |
| --- | --- | --- |
| G0002 | 调查对象编号:  Respondent ID: | 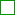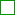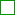 |
| G0003 | 是初次调查还是再次调查？ 1.初次 2.再次  Is this the initial or retest interview? 1. Initial 2. Retest | 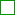  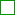 |
| G0004 | 如果是再次调查，请写出两次调查间的间隔天数：(天)  If retest interview, indicate number of days between initial and retest: (Days) | 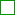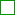 |
| G0005 | 调查员编号  Interviewer ID | 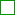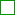 |
| G0006 | 调查员姓名  Interviewer name |  |

长治医学院预防医学系

2014.01

第一部分：基本情况

Part I: General Information)

|  |  | **填写答案处** |
| --- | --- | --- |
| G1001 | 姓名(请用汉语拼音填写)Full name(please fill in English) |  |
| G1002 | 性别: 1-女性 2-男性 Sex:: 1-Female 2-Male | 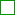 |
| G1003 | 年龄 (岁) Age (yrs) | 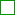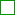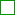 |
| G1004 | 民族(请用汉语拼音填写) Nationality (Please fill with English) |  |
| G1005 | 最高学历: 1-小学以下 2-小学 3-初中 4-高中 5-大专/大学 6-硕士 7-博士  The highest level of education: 1-Less than primary school 2-Primary school completed 3-Secondary school completed 4-High school (or equivalent) completed 5-College / pre-university / University completed 6-Master degree 7-Doctor degree | 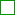 |
| G1006 | 职业: 1-公务员 2-专业技术人员 3- 企业管理人员 4-工人5-农民 6-在校学生 7-服务行业人员 8-自由职业者 9-其他  Current job: 1-Government employee 2-Professional or technician 3-Enterprise managers 4-Blue-collar worker 5-Farmer 6-Student in school 7-service worker  8- Freelance 9-others | 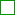 |
| G1007 | 个人月收入: 1-999元以下 2-1000~2999元 3-3000~4999元 4-5000元以上  income per month: 1-less than 999 Yuan 2-1000~2999 Yuan 3-3000~4999 Yuan 4-5000 Yuan or above | 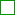 |
| G1008 | 婚姻状况: 1-已婚 2-未婚  Current marital status: 1- Married 2-Unmarried | 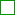 |
| G1009 | 体重(公斤) Weight(kg) | 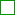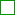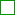 |
| G1010 | 身高(厘米) Height(cm) | 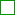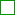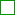 |

注：每个空格处填写一个数字。横线处请用大写字母填写。

第二部分：健康保护行为量表

Part Ⅱ: Health Protecting Behavior Scale

本调查是对您自身健康行为的了解。获得这些信息将有助于追踪你日常行为和生活习惯变化。请回答所有问题，在方框内填下你所选择的数字。如果你对答案不确定，请给出你认为最接近的答案。

This survey asks for your behavior about your health. This information will help keep track the change of your general acts and habits. Answer every question by marking the answer as indicated. If you are unsure about how to answer a question, please give the best answer you can.

PART1

1. 我知道自己的血压值know the value of blood pressure 1-是yes 2-否no
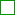


2. 我知道自己的血糖值know the value of blood sugar 1-是yes 2-否no
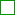


3. 我定期进行健康体检do [physical](javascript:void(0);) [examination](javascript:void(0);) regularly 1-是yes 2-否no
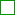


4. 我的收入足够生活上基本消费 Income enough for general consumption
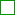


1-是yes 2-否no

5. 我主动学习应对灾害、突发事件的方法learn method coping with disaster and emergency 1-是yes 2-否no
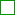


PART2

6. 在日常饮食中使用植物油代替动物油

replacing animal fat with vegetable oil
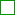


1-从不never 2-很少rarely 3-一半时间general 4-常常usually 5-总是always

7. 在日常饮食中控制食盐的用量control salt
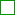


1-从不never 2-很少rarely 3-一半时间general 4-常常usually 5-总是always

8. 在日常饮食中限制糖类的用量control sugar
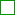


1-从不never 2-很少rarely 3-一半时间general 4-常常usually 5-总是always

9. 每天吃新鲜的蔬菜半斤到一斤eat vegetable every day 250g-500g
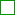


1-从不never 2-很少rarely 3-一半时间general 4-常常usually 5-总是always

10. 每天吃半斤左右的水果eat fruit every day about 250g
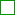


1-从不never 2-很少rarely 3-一半时间general 4-常常usually 5-总是always

11. 我超过正常体重时就控制体重keep weight
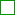


1-从不never 2-很少rarely 3-一半时间general 4-常常usually 5-总是always

12. 我担忧食品安全的现状worry for food safety
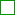


1-从不never 2-很少rarely 3-一半时间general 4-常常usually 5-总是always

13. 我每天参加中等强度的活动且时间大于30分钟
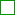


do physical activity every day 30 min or more

1-从不never 2-很少rarely 3-一半时间general 4-常常usually 5-总是always

14. 我劝说周围的吸烟者戒烟persuade other to quit smoking
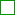


1-从不never 2-很少rarely 3-一半时间general 4-常常usually 5-总是always

15. 周围有人吸烟时，我会尽量离远be far from smoking
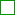


1-从不never 2-很少rarely 3-一半时间general 4-常常usually 5-总是always

16. 我有充足的睡眠get enough sleeping
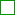


1-从不never 2-很少rarely 3-一半时间general 4-常常usually 5-总是always

17. 生病后我听从医生的用药指导take doctors guide for medicine
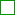


1-从不never 2-很少rarely 3-一半时间general 4-常常usually 5-总是always

18. 我丢弃过期的药品discard drug out of date
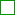


1-从不never 2-很少rarely 3-一半时间general 4-常常usually 5-总是always

19. 清楚对近亲结婚的危害know harm about intermarriage
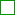


1-从不never 2-很少rarely 3-一半时间general 4-常常usually 5-总是always

20. 我在驾驶或乘坐汽车时，按规定正确使用安全带use seat belt
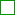


1-从不never 2-很少rarely 3-一半时间general 4-常常usually 5-总是always

21. 我使用工作场所的保护措施use protection measures in workplace
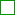


1-从不never 2-很少rarely 3-一半时间general 4-常常usually 5-总是always

22. 在强烈阳光下，保护皮肤，穿合适的衣服、使用防晒护肤品或遮阳伞
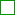


Protect skin under sunshine

1-从不never 2-很少rarely 3-一半时间general 4-常常usually 5-总是always

23. 我使用净水设备（净水器、纯净水）use water purifying plant
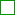


1-从不never 2-很少rarely 3-一半时间general 4-常常usually 5-总是always

24.在雾霾、风沙天气我佩戴口罩 wear a mask in the hazy or wind weather
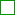


1-从不never 2-很少rarely 3-一半时间general 4-常常usually 5-总是always

25. 我能够适应新的生活、学习和工作环境easily adapt to a new environment
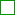


1-从不never 2-很少rarely 3-一半时间general 4-常常usually 5-总是always

26. 我空闲时间可以享受到乐趣enjoy the pleasure at free time
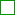


1-从不never 2-很少rarely 3-一半时间general 4-常常usually 5-总是always

27. 我能自我放松和自找乐趣self-relaxation
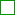


1-从不never 2-很少rarely 3-一半时间general 4-常常usually 5-总是always

28. 我从他人那里得到所需要的帮助get help from others
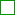


1-从不never 2-很少rarely 3-一半时间general 4-常常usually 5-总是always

29. 我乐于采纳别人的建议take other’s advice [pleasureably](javascript:void(0);)
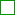


1-从不never 2-很少rarely 3-一半时间general 4-常常usually 5-总是always

30. 在一些关键时刻我会紧张，但能很快镇定下来keep calm in some key moment
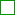


1-从不never 2-很少rarely 3-一半时间general 4-常常usually 5-总是always

31. 当我焦虑不安时，我通过做其它事情来改变这种心情
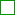


Do something to change anxiety

1-从不never 2-很少rarely 3-一半时间general 4-常常usually 5-总是always

32. 当遇到困难时，我想办法去解决它try best to solve problems
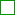


1-从不never 2-很少rarely 3-一半时间general 4-常常usually 5-总是always
